# The COVID-19 Pandemic Related Lived Experiences of Individuals With a Spinal Cord Injury/Disease

**DOI:** 10.3389/fresc.2022.834909

**Published:** 2022-04-05

**Authors:** Ethan Simpson, William C. Miller, Julia Schmidt, Jaimie Borisoff, W. Ben Mortenson

**Affiliations:** ^1^Department of Occupational Science & Occupational Therapy, Faculty of Medicine, University of British Columbia, Vancouver, BC, Canada; ^2^GF Strong Rehabilitation Research Program, Vancouver, BC, Canada; ^3^International Collaboration on Repair Discoveries, Vancouver, BC, Canada; ^4^Rehabilitation Engineering Design, British Columbia Institute of Technology, Burnaby, BC, Canada

**Keywords:** COVID-19, spinal cord injury, phenomenology, experiences, mobilities paradigm

## Abstract

**Introduction:**

COVID-19 related restrictions and recommendations have impacted everyone. Those living with a disability, such as individuals with a spinal cord injury (SCI), may have had pandemic related changes made yet more challenging by societal failures to accommodate their mobility, physical abilities, and health care needs. To better understand participants experiences we drew upon Heidegger's phenomenology and the mobilities paradigm. The objective of this study was to explore COVID-19 pandemic related lived-experiences of individuals with an SCI.

**Materials and Methods:**

This study used an interpretive phenomenological methodology. Semi-structured interviews were the primary means of data collection. These were conducting in May and June of 2020, roughly 2–3 months into the pandemic. Transcript data were analyzed using a phenomenological methodology.

**Results:**

We interviewed 22 participants with SCI, the mean age was 54 years, and nine were females. We identified three themes: (1) *Experiencing changes to mobility and daily life* described how new rules had impacted everyday life and usual routines, particularly in regard to mobility. (2) *Struggling with new challenges* explored some of the negative experiences of the pandemic. (3) *Being resilient in the face of a new normal* conveyed the resilience participants exhibited despite challenges.

**Conclusion:**

Although our findings indicate some positive changes and highlight the strengths that many individuals with SCI have, they also accentuate issues with ableism within the medical system. Certain changes were made primarily because people without disabilities needed them, and several COVID-19 changes were made without consulting individuals with disabilities. With physical movement restricted, our findings emphasize the importance of the movement of information and a need for increased dialogue with people in the SCI community about their ongoing pandemic related needs.

## Introduction

COVID-19 related restrictions and recommendations have changed the way individuals interact with one another and engage in their typical activities. Generic recommendations have urged individuals to maintain physical distance of at least six feet, to wear a face mask when in public, to wash hands regularly using soap and water or hand sanitizer, and to regularly disinfect and clean surfaces (1).

In addition to implementing global recommendations, Canada and its provinces implemented multiple precautionary measures. Although there were minor discrepancies between provinces as to the timing, there was a consensus in the type of measures implemented. The first phase of British Columbia's COVID-19 response aligned with a declaration of a public health emergency and consisted of mandated physical distancing, banning mass gatherings, closing non-essential personal services, reducing in-classroom learning, restricted visitations to health care and assisted living facilities, postponing non-urgent surgeries, closing provincial parks, and requiring all visitors arriving from abroad to develop and adhere to a 14-day isolation plan (2). There has been concern of the potential for compounding issues stemming from the implementation of such measures.

A key population of concern that has received less attention during the pandemic are those living with a disability, such as spinal cord injury (3). Disabilities cover a broad spectrum of conditions, and the management requirements and risks vary vastly. A public health article indicated that examples of concerns to this population include service provision, transportation, and accessibility to both facilities and information. Individuals with disability may rely on service provision for personal care, medication, and food but this has been made difficult by the requirement to physically distance/isolate and the need for PPE that is not readily available. There may also be inequities regarding access to information and services, potentially due to physical, linguistical, or technological barriers (4). According to a health sociology review, inequities extend to ableism with adoptions of a one-size-fits-all model to healthcare in certain countries indicating a lack of consideration toward disability needs (5). Ableism can be defined as “stereotyping, prejudice, discrimination, and social oppression toward people with disabilities (6).” Concerns of individuals with disabilities included whether they would be offered the same level and access to care as awarded to people without disabilities.

There is very limited research about the concerns or difficulties of individuals with spinal cord injuries related to COVID-19. A survey distributed to clinicians who care for individuals with spinal cord injuries identified potential concerns (7). These included whether there would be sufficient caregivers still available, whether they would be able to get access and maintenance for specialized medical care and equipment, whether they would be able to get to appointments, and about a potential inability to even self-quarantine if desired or required (7). An editorial relating to spinal cord injuries and the first wave of COVID-19 suggested that the requirement for most individuals with spinal cord injuries to use some form of wheelchair can make physical distancing more difficult (8). There is also a fear of having to attend hospitals, but non-attendance could lead to greater complications of untreated conditions. Attempts to address some of these issues include the increased use of telehealth to reduce the need for in-person appointments with doctors and physical therapists (9).

### Theoretical Framework

To explore the experiences of individuals with spinal cord injuries during the COVID-19 pandemic, we drew upon the mobilities paradigm (10). The mobilities paradigm challenges the static or sedentary focus of many other theories, acknowledging the importance of movement for aspects of life such as work and leisure. The work of Merleau-Ponty on the phenomenology of movement viewed movement as the link between the body and the world, with life being the unfinished act of moving into and creating the space in which we encounter others and the world (11). The mobilities paradigm does not suggest that mobility is a new phenomenon but rather acknowledges movement as a central point of inquiry (12). It focuses on the movement of people, information, and objects, along with the implications of said movements (10). The mobilities paradigm acknowledges that mobility itself can both reflect and reinforce power, with not all having equal ability for movement (10). This indicates that the implications of the implemented measures will vary between individuals and societies. Drawing upon this theory will allow us to gain a greater understanding with the aim of this study to explore the COVID-19 pandemic related lived experiences of community dwelling individuals with spinal cord injuries.

## Materials and Methods

This study is part of a larger study exploring COVID-19 experiences across a range of populations, including disabilities and families (13). This study used an interpretive phenomenological methodology (14). Heidegger's interpretive phenomenology explores the lived experience of participants relating to a given phenomenon. In particular, its rich philosophical tradition and its allowance of the use of theoretical orientations is congruent with and complements the mobilities paradigm (14). Qualitative data will be reported using the Consolidated criteria for Reporting Qualitative research (COREQ) checklist (15). Ethical approval was received from the University of British Columbia and Vancouver Coastal Health (H14-01737).

### Participants

Participants (1) were Canadian, (2) were 19 years old or more, (3) were comfortable with written and spoken English, (4) had access to technology and internet, and (5) had a spinal cord injury/disease. Individuals were excluded, following a short screening interview, if they have moderate to severe cognitive impairment that impaired internet use. We used convenience sampling and participants were recruited through Vancouver Coastal Health e-blasts and online postings at the International Collaboration on Repair Discovery, by using a list of previous research participants who have consented to being contacted for future studies, by using study researchers' social media pages, and through word of mouth.

### Protocol

Participants who expressed interest in participating in the study were sent a link containing more details of the study and an option to provide their informed consent electronically. Individuals unable to provide informed consent electronically had the opportunity to do so verbally over the phone. Along with verbal consent, they were asked to print, sign, and mail a consent form to the rehabilitation center. The forms were verified by a research assistant prior to data collection.

Data were collected by conducting semi-structured interviews using a videoconferencing platform. Zoom was deemed an appropriate videoconferencing platform given its ease of use, security, cost-effectiveness, and data management options (16). The interview guide was developed by authors ES, WCM, and WBM. The full interview guide can be found in the Supplementary Materials, but example interview questions include:

“What has your experience of COVID been like?” “What activities are you doing to connect with others?”

Participants were given the opportunity to share photographs relating to their experiences during the interviews in order to facilitate deeper discussions. Interviews were audio recorded and subsequently transcribed. Following each interview, participants were provided an honorarium of c$30.00.

### Analyses

Analyses of the transcripts were performed in Microsoft Excel and Miro, and followed an explicitation process used in the phenomenological methodology outlined by Groenewald. The process has five phases: “bracketing and phenomenological reduction,” “delineating units of meaning,” “clustering of units of meaning to form themes,” “summarizing each interview, validating it and where necessary modifying it,” and “extracting general and unique themes from all the interviews and making a composite summary” (17). We had four researchers, all trained by corresponding author WBM, familiarize ourselves with a subset of the transcripts and discuss the initial extraction of meaningful units. Researcher ES then spearheaded the delineating of units from subsequent transcripts while having regular meetings with the other researchers to discuss clustering and formation of themes. The formation of themes from the codes can be seen in Table 1.

**Table 1:** Development of themes.

**Theme**	**Sub-theme**	**Code**
Experiencing changes in mobility and daily life	Experiencing changes to normal routine	Experiencing changes in work-life and education Experiencing changes to normal routine Experiencing changes with daily chores Experiencing changes with travel and transportation Experiencing time differently during the pandemic
	Adjusting to changes in health care	Adjusting to medical system changes Having to adjust to changes in home care
Struggling with new challenges	Feeling frustrated and anxious	Feeling anxious about the foreboding situation around them Feeling anxious about themselves
	Struggling with reduced social interaction	Having frustrations inside the home Having frustrations outside of the home Experiencing changes with socializing Feeling socially isolated Feeling there are limited recreational activities
Being resilient in theface of a new normal	Managing new information	Considering planning and preparing essential Staying up to date with potentially stressful information Trying to follow COVID-19 precautions and guidelines
	Personal coping	Trying to stay mentally active Trying to stay physically active Trying to stay positive
	Connecting with others	Empathizing with others and being mindful Expressing enhanced gratitude and appreciation Intentionally connecting with others Offering and providing emotional support and help to others Receiving and being offered emotional support and help by others

We employed trustworthiness strategies that align with our chosen constructivism paradigm by using Morrow's transcendent criteria: social validity, subjectivity and reflexivity, adequacy of data, and adequacy of interpretation (18). For example, for subjectivity and reflexivity, we acknowledge that research is subjective, but we embraced the positioning of multiple researchers when coding transcripts to provide complementing perspectives, and not dismiss dissenting ideas, ultimately to enrich our interpretation. In alignment with constructionism, interpretive phenomenology does not attempt to use bracketing to remove the role of the researcher but rather embraces them as an integral co-constructor of meaning. It was important, however, to be cognisant of our positioning and biases. Interviewers were asked to review the interview guide before conducting any interviews to gain an understanding of pre-conceived responses we may be expecting. This helped us to avoid supporting our own biases and we also recorded interview reflections following each interview. Of the authors, all are Caucasian, four are male, and one is female. Three have a background in Occupational Science, one has a background in engineering, and one has a background in kinesiology. One author has lived experience with an SCI.

Regarding adequacy of data, in qualitative research there is no set number as to how many interviews or participants are required because the insights generated are largely dependent on the richness and variety of each interview and the capabilities of the researchers. Some propose that data should be collected until the point of redundancy when no new information is being gained from additional data collection. Redundancy is related to saturation which similarly is defined “that no additional data are being found whereby the sociologist can develop properties of the category” (19). Essentially, they deem the category to be saturated. This description can be misleading, however, as it may indicate a false sense of completion. A more appropriate term may be that of sufficiency where there is sufficient depth of understanding to provide and interpretation of the phenomenon (20). Guest et al. (21) have recommended that this can be achieved with as few as 12 interviews, which we exceeded for this study.

## Results

The demographic information of our sample is outlined in Table 2. It consisted of 22 individuals with spinal cord injuries, with a mean age of 54 and 9 of whom were female.

**Table 2:** Sample demographic information (*N* = 22).

**Demographic factor**	**Responses**	**X ±SD** **or *n* (%)**
Age		53.77 ± 11.07
Birthplace	Canada	14 (64)
	Other	6 (27)
	PNA	2 (9)
Sex	Female	9 (41)
	Male	12 (55)
	PNA	1 (5)
Live in assisted living	Yes	0 (0)
Live alone	Yes	7 (32)
Provide care	Yes	2 (9)
Receive care	Yes	8 (37)
Education	Graduated from high school/GED	1 (5)
	Some or graduated college/trade school/university	18 (82)
	Some or graduated post-graduate school (i.e., PhD, MSc)	3 (14)
Household income	Less than $14,999	2 (9)
	$15,000 to $44,999	5 (23)
	$45,000 to $74,999	6 (27)
	Greater than $75,000	3 (14)
	PNA	6 (27)
Employment status	Employed full-time	3 (14)
	Employed part-time	3 (14)
	Self employed	2 (9)
	On disability assistance	7 (32)
	Retired	5 (23)
	Unemployed	1 (5)
	Other	1 (5)
Time with disability	Since birth	2 (9)
	Since childhood	1 (5)
	Since adolescence	4 (18)
	Since adulthood	6 (27)
	Later in life	9 (41)
Type of injury	Complete	7 (32)
	Incomplete	14 (64)
	PNA	1 (5)
Diagnosis	Paraplegia	10 (45)
	Tetraplegia	6 (27)
	Other (e.g., spina bifida)	6 (27)
Tested positive for COVID-19	Yes	0 (0)

*PNA, prefer not to answer; provide care, the participant provides care (assistance with daily activities) to someone they live with; receive care, the participant receives care from someone they live with*.

We identified three themes that are encompassed by an overarching theme: *trying to manage and stay healthy during a pandemic*. The three themes are: *experiencing changes to mobility and daily life, struggling with new challenges*, and *being resilient in the face of a new normal*.

### Experiencing Changes to Mobility and Daily Life

The first theme, *experiencing changes to mobility and daily life*, described how restrictions and guidelines stemming from the COVID-19 pandemic had influenced typical daily life, especially in terms of participants' mobility. Under this, we identified two sub-themes: *experiencing changes to normal routine* and *adjusting to changes in health care*.

The first sub-theme, *experiencing changes to normal routine*, identified aspects that had been altered such as chores, work-life, education, travel, transportation, and time. A 40-year-old female with paraplegia explained how the basic activity of grocery shopping has been impacted by COVID-19:

There's like 250 [people] allowed in a grocery store at once. So Costco's all lined up outside Wal Mart was lined up outside the other day. No, I'll just wait, I'm not going when it's busy (P6).

Attempts to adjust to such changes would largely require adjustments to typical movement behavior, such as going shopping at different times than usual.

With movement restricted, all participants expressed a desire to travel again. For some, such as a 54-year-old male with paraplegia, the desire to travel is based on leisure and seeing family and friends:

I think that as soon as there is a portal across the border to the US, that opens up a whole bunch of places for me to drive to. Because I have friends in [Location], I have friends in [Location], and so it would an opportunity for me to go and visit my friends. Protecting myself and them, as much as possible, of course, in the process. But at least I will not be stuck in [Location] (P3).

For others, such as a 71-year-old male with incomplete paraplegia, travel also allows them to engage in their hobbies and projects:

Once this virus is over, we're actually […] launching a podcast, which will be done by us, myself my wife at various resorts and hotels around the world from the perspective of accessibility using drones and camcorders and stuff like that, we did one already in Manila and we did one in Cancun, Mexico, and they were well received so we're just waiting until this pandemic decides to go away, and then we're off and running (P24).

In other aspects of life, a few participants experienced changes in work-life. For those still in employment, some adjusted well to the new methods, such as a 50-year-old female with quadriplegia reporting her transition and not needing to commute:

So luckily, most of my work is when the students aren't in school. Most of my work is on the computer, so I was able to transition pretty easily, so that was nice. I had a vice principal that I called and just told him the things that I wanted from work, and he brought them to me in a box (P12).

Others were less fortunate, such as a 46-year-old male with incomplete tetraplegia describing the effect of COVID-19 on their job:

I also score keep minor hockey teams, tournaments, but that would be in the spring and they all got canceled. Hockey's a lot of money and because my money is cash, I get no [financial support] or anything (P2).

Work-life could be impacted by factors such as children doing school from home, as experienced by a 40-year-old female with paraplegia:

The kids kind of hang out in their own rooms, so that means that helps. They're doing their schoolwork. […]. So I mean, the kids have their rooms. I work during the day, so I hide in my office. If my husband's home and not working, he hides and takes over the entire living room, so that's kind of annoying, but I mean, he doesn't really have anywhere else to go (P6).

The second sub-theme, *adjusting to changes in health care*, related to challenges with caregivers and adjusting to medical system modifications. A key issue regarding caregivers was their availability. A 53-year-old female reported:

So what happened was when this COVID thing started, they had a real shortage of home support workers, and […] It was stressful for me. Luckily, I'm married and there's one person in my house, well mind you he works, […] but, […] that was pretty scary (P14).

Some participants felt fortunate that they were able to maintain their caregiver during COVID-19 such as a 54-year-old male with paraplegia:

I was able to... very fortunate for me. Find an attendant who was willing to come in during the pandemic with all kinds of precautions…masks, washing the hands and everything else. And so I've had an attendant and we have been able to take care of our own things since my wife is not paralyzed. And therefore, when there's stuff to be done around the house and if my attendant is not there, she does it (P3).

As their mobility is already restricted due to their spinal cord injury, most participants appreciated the convenience associated with a shift to telehealth appointments, such as a 53-year-old female who felt it was an easy solution:

With my doctor, I do have to see her in person at times. But honestly, why do I have to go all the way to Vancouver and back? It's not good for the environment, it costs money, whatever, when it's a simple thing. […] With technology, I've had two appointments with my doctor this way, and they were easy, easy solutions (P14).

While still viewing the change as a positive, there was some frustration that the change had not been made sooner:

All the stuff that people with disabilities have been advocating for two decades, being able to work from home, medical appointments from home, financial assistance. All that stuff, boom, came out in 6 weeks. Done (P18).

Overall, the shift to telehealth appears to have been positive but there are still challenges for in person appointments and frustrations it took a pandemic for the change to come into effect.

### Struggling With New Challenges

The second theme, *struggling with new challenges*, explored certain negative consequences associated with the pandemic and included two sub-themes: *feeling frustrated and anxious* and *struggling with reduced social interaction*. For some individuals, such as a 58-year-old female with tetraplegia, the compounding impacts of the pandemic reminded them of their disabilities:

So yeah, I think it's definitely one of those things that's […] reminded me that I actually do have a spinal cord injury because I think a lot of days I […] have lived the last 23 years […] not thinking that way (P1).

Regarding the first sub-theme, participants experienced a mix of frustrations both in- and outside of the home. The frustrations were often compounded by a reliance on the cooperation of others due to personal mobility constraints. Within the home, tensions could arise when a household contained varying views such as a 40-year-old female with paraplegia who expressed this concern:

I think that it should still be thinking seriously. And I think, my husband is starting to lean toward the more less serious side. And being that I have all these health things that have been wrong with me in the past, I don't know if I'm more susceptible to it or not. […] the information that comes out with that every day is changing, and you never know. Right? It's hard when you have differences in opinion that you can't necessarily agree (P6).

Outside of the home, frustrations primarily related to people around them not adequately following guidelines. A 35-year-old female with spina bifida stated:

I don't know whether some of it is ignorance or just people being selfish, […] or not understanding that […] what they do affects other people. I had [..] friends of mine that […] just don't care, on ferries visiting their people […] and stuff like that and just […] not wearing masks or not sanitizing or thinking that masks will protect you. […] just frustrates me (P7).

The stark change in lifestyle and accompanying uncertainties brought a share of anxiety. Triggers of anxiety varied between participants, for some it was predominately the health aspect with fear of contracting the virus itself. A 66-year-old male with paraplegia expressed many of these concerns:

I'm afraid that I won't get my medications. […] And so I think this is something for all people with spinal cord injuries is that we have immediate concerns about our care whether it's our caregivers coming in, getting COVID from our caregivers, and so that's a concern for all of us. And the other thing with people like me, not just people with spinal injuries but […] because we are dealing with spinal cord injuries is that if we get this, we are going to die. I mean chances are, we will die. […] So those are real, what would you want to call them, those are facts about COVID 19 that people with disabilities have to come to terms with (P4).

For other participants anxiety was related to the broader societal implications of COVID-19. These included impacts to the economy, to industries, and to socializing. In particular, concerns often related to the broader impact of the restricted movement of humans, goods, and services within and between countries. A 58-year-old male with a spinal cord injury expressed concern regarding the future:

There could be a big hit to Canada, both from the American side and the Canadian side a lot of exports gets traded in two countries, that trickles down to my job. Affect us economically, socially Yeah, […] unless a lot of money gets thrown out, still a lot of pain to go job wise, can't operate at this capacity, […] that's the toughest part of this, is the uncertainty (P17).

There were also concerns about their equipment, as were voiced by a 48-year-old male with quadriplegia:

One of the most important things is, is my equipment gonna break down? And if it, when it does, is the people that are repairing it going to do it on a timely basis. And I was very worried about that and touch wood. Nothing broke out. So that all worked out (P23).

Anxieties may have manifested themselves into altered behaviors and mood. A 51-year-old female with a spinal cord injury experienced reduced sleep and altered mood:

Not sleeping great. Definitely, I would think some days my mood's a bit altered. […] I've kind of had days where just, you know, off, I guess. I think I'm snacking a whole lot more. […] I think that when you're home more you tend to eat more of the foods you probably shouldn't eat, but you're home, and you're bored, so you snack more (P9).

In addition to mood, a 33-year-old male expressed how mental states such as motivation were impacted:

What I feel like in my mind I'm motivated to find out about this stuff, but I'm not motivated to actually make, like, the YouTube channel I thought would work, you know? Which is weird, normally I'm really motivated to do this stuff (P19).

The changes in mood may have also been related to changes in social dynamics.

The second sub-theme, *struggling with reduced social interactions*, explored the responses and consequences of physical distancing requirements, limiting the desired movements of people. In some cases, participants compared restrictions with imprisonment. For example, a 50-year-old male with paraplegia indicated:

I say I almost feel imprisoned […] Probably imprisoned. […] Oh, I wouldn't say I feel abandoned because that would be the other parties, you know, not wanting to see me. So couldn't say abandoned. I would say probably imprisoned is a better word. Not of my own doing (P20).

This lack of interaction led to some participants, like a 69-year-old female with spina bifida, feeling isolated at times:

Yeah. I mean, the more negative is that. I'm not socializing as much as I was before and […] and I feel isolated (P13).

Not only was the lack of interaction and restricted mobility in itself troubling, but for some it reduced their ability to deal with other sources of stress. A 48-year-old male with quadriplegia struggled with the impact on their coping strategies stated:

I think because of the things that I've been through in the last 6 years, I'm kind of thriving as a quadriplegic because I'm very strong mentally, But what was difficult and I was articulating this to my caregiver. […] part of my coping mechanism is the plethora of friends, I'm lucky to have a family that I visit almost on a daily basis, and also just driving around my lovely neighborhood where I live here by the waterfront. […] when I didn't have those for about 3 weeks, it was it was starting to, I was starting to get wound up by that, I need to get out of the house and talk to someone else. […] And, of course, when they're not there, you have to just try to rely on yourself (P23).

In this case, the participant struggled with the limited social interaction and not being able to get out into the community as much.

### Being Resilient in the Face of a New Normal

Participants conveyed substantial resilience to the impacts of the pandemic and its movement restrictions. For most, this resilience was established through their past experiences, as indicated by a 67-year-old male with tetraplegia:

Well, if they're spinal cord injury, look at what you've already gone through. This is not that bad [laughs]. Basically, you've already managed to survive this storm. You're going to go further (P16).

Within this theme, we identified three sub-themes: *managing new information, personal coping*, and *connecting with others*.

In the first sub-theme, *managing new information*, with restrictions and recommendations ever evolving, participants tried to understand and apply new information. One aspect of this was finding reliable information in the first place with the movement of appropriate information not always satisfactory. A 51-year-old female with paraplegia indicated how this could be difficult:

Facebook is just maybe post something that I found interesting, or to read what my nieces and nephews are doing, […] There is so much garbage out there, so many people get their news from Facebook, like really, you know? There is so much news information. Search for COVID you really see it, right? All this crappy, I think people started with good intentions, but its misinformation, right? And I don't continue on those chains or anything like that (P8).

There were, however, general sources that some people did trust including the one outlined by a 33-year-old male with an incomplete spinal cord injury:

Like [our Public Health Officer] What helped was her press briefings because it kind of told me, like, how careful do you need to be? And you could read her language and could read the numbers. And she's like, uh oh, boy, numbers are high. I'm like, ok, stay home. but if she's like, we can start thinking about opening up, I'm like, okay, so there's something in the numbers that makes her think that and yeah, I trust the science basically (P19).

When applying the information, a 45-year-old male with incomplete tetraplegia indicated how they follow safety recommendations:

Don't panic. Follow the basics like still, when people come in, to wash their hands, I don't make them take off their shoes and put on a mask anymore. Um, so you have to calculate your level of risk, but we know more now (P18).

This could, however, be difficult at times due to functional abilities and associated requirements. A 33-year-old male with an incomplete spinal cord injury described how his use of a wheelchair, combined with cleaning agents selling out, impeded his mobility:

In the early days, […] nobody really knew a whole lot about this virus. Um, like nobody really knew how it spread. Nobody knew how long it lingered on surfaces, which was my main concern, because I have to touch everything. […] And everyone was buying up all the stuff that would otherwise help people with wheelchairs like, you know, many of us use wipes and sanitizer and all that. So, a lot of that went really quickly, um, which sucked because it, it meant that we couldn't go anywhere even if it were safe (P19).

Participants would find their own ways of coping with these complications.

The second sub-theme, *personal coping*, displayed participants' individual strategies to overcome some of these additional challenges. An aspect of the resilience piece is the mindset to stay positive, as indicated by a 67-year-old male with tetraplegia:

There's nothing that will get me down. Now emotionally I'm in good spirits (P16).

Resilience was facilitated by using previous experiences. A 53-year-old female used past experiences to help her cope:

I guess I just keep coming to that, 10 years of cognitive behavioral therapy after that accident taught me a lot about managing stress and anxieties and ways of coping. I have that behind me. […] What doesn't kill you makes you stronger, but not for everybody, but for some people. Definitely. I'm not saying I appreciate all the things that I've been through, […] But, hey, it does build resilience (P14).

Participants have found it important to stay both physically and mentally active during the pandemic with so many of their usual activities rendered unavailable by movement restrictions. A 62-year-old male with paraplegia explained how they stayed active:

I had more time to do weights, so I was doing weightlifting exercises just in my home area at home. I've got some weights there. Some of it's aerobic, and some of it's weightlifting. So I was doing that. Pretty quick in the COVID situation we lost our ability to participate in group sports, so my wheelchair basketball stopped. […] I've been able to get out of my bike, which has been a great help. And just recently, in the last 2 weeks, I've been able to get out of my kayak. I'm doing biking and kayaking. That's what I do right now is my main exercise. I've stopped the weight training (P10).

Ways that participants stayed mentally active included reading, doing online courses and, a way that a 40-year-old female with paraplegia stayed mentally active included, doing crosswords.

The third *sub-theme*, connecting with others, identified social strategies for coping with restrictions. A big aspect of this was finding ways to stay connected in wake of the need for physical distancing. A 58-year-old female explaining how she has been staying connected:

So as far as social stuff outside of the house […] we've kinda been doing the distancing, so other than a few visits in the car, stopping by, and doing that kind of visits, really haven't seen family either, to be honest. It's mostly Zoom calls, FaceTime stuff, and text […] the last week or so I've started venturing out and kinda going for some distanced walks just around the neighborhood with another friend who's not working at the time as well (P1).

This shift to primarily using technology has allowed a means of connectivity without the need to physical move. It has also led to broader outreach by some individuals, including a 62-year-old male with paraplegia:

Oh, I've made connections with a lot of people that I haven't talked to for a while. It's almost an excuse to text somebody or to phone somebody up or facetime somebody. I haven't made contact in half a year, a year, you know, with family that's far away. I've been doing some video chats with them and I've never done that in my life (P10).

Despite the upheavals associated with COVID-19, participants indicated having an increased sense of gratitude during the pandemic. A 48-year-old male with quadriplegia expressed:

One of the big things with COVID for myself and many of people in my neighborhood and my friends is we're all so much more thankful for being here, you know, and appreciate the basic things in life. And I think during times of struggle, if you could take those important things as a message, that can give you the calmness you need during that time (P23).

A 51-year-old female with paraplegia explained how this was augmented by the social support of helping each other in times of need:

We didn't go out, and found a volunteer shopper. And some neighbors offered to help, but most of my neighbors are like still working. One guy wasn't working, so we got him to pick up groceries 1 day, cause he was going. But, right away, a lot of groups and organizations I belong to, they were really good at getting online stuff up pretty quickly, and communicating all the COVID information (P8).

This demonstrates how neighbors worked together to help each other.

## Discussion

There has been limited research exploring the lived experiences of individuals with spinal cord injury during the COVID-19 pandemic. Our results illustrate the changes to their daily lives and movement patterns and the compounding impact that restrictions have had on them. We have organized our discussion based on the themes we developed.

### Experiencing Changes to Mobility and Daily Life

Not surprisingly, the first theme identifies various ways in which everyday life has changed since pre-COVID-19. Relatively few participants described a switch to working from home. With 50% of our working age sample unemployed, this aligns with broader Canadian data, reporting that 59% of working age individuals are unemployed 5 years post-spinal cord injury and 72% receive some form of financial compensation (28). Employment and mobility are inherently connected with work related travel becoming inseparable from the economic activity (26). If mobility is impeded post-spinal cord injury, this may limit job options available. A lack of employment can therefore be attributed to mobility inequities as well as a clear systemic issue. Those with spinal cord injury who return to work often end up in less secure positions when seeking competitive employment (29). As indicated by employment, the challenges and impact of changes could be confounded during the COVID-19 pandemic for individuals with spinal cord injury.

In addition to complying with new rules, everyday tasks such as grocery shopping and public transportation are already complicated for this population. There is a clear disparity in mobility in individuals with spinal cord injury compared to people without disabilities. This can impose a lack of independence due to basic accessibility barriers. For example, the ability to get on a bus with a wheelchair may be inhibited by inaccessible bus stops and buses (30), and for shopping a study with spinal cord injury participants in Massachusetts found that 60% of participants reported issues with grocery shopping (31). While largely inconvenient, however, there were some silver linings within these changes.

The widespread adoption of telehealth addressed a long-needed request of the spinal cord injury community. Individuals with spinal cord injury face multiple barriers when it comes to accessing health care, including geographical, physical, and transportation (22). The use of telehealth removes the requirement for the individual to move, enabling a wide variety of clinical issues to be addressed without the need for the individual to leave their place of residence. Despite previous research showing clear benefits and efficacy of telehealth for individuals with spinal cord injury, it was the COVID-19 pandemic that prompted its wide-spread adoption as opposed to just gradual increases. This change enabled individuals with spinal cord injury, but still highlights issues with ableism within the healthcare system as it was the needs of people without disabilities that incited the change (32).

In Western cultures, those deemed independent are often preferentially enabled (33). This emphasis on independence can lead to less funding and services for those with disabilities (34). This bias develops a negative feedback loop (35). For example, issues with mobility, potentially relating to a disability such as spinal cord injury, can restrict the ability to perform daily activities and move between places. These restrictions are due to the way society is organized but continue to exacerbate and limit the opportunities available. Ultimately broader forms of mobility, such as access to health care, education, and employment, may be impacted by society's response to disability. During the pandemic, the risk of inequities regarding access to healthcare for individuals with disability has been a concern from the onset (4). The concern spreads to whether systemic bias would see lifesaving medical equipment withheld from individuals with disabilities in the event of a shortage (7). Despite policies to prevent exactly this, there have still been reported cases where disability discrimination may have factored into healthcare. For example, it is thought that Michael Hickson, who was paralyzed, did not receive appropriate care in his COVID-19 course that concluded in death as one of his physicians was quoted as saying, “right now, his quality of life—he doesn't have much of one,” simply because Michael Hickson was paralyzed (24). There is an ableist assumption that individuals with a disability have a lower quality of life and this pandemic has further exposed that belief (36). This belief runs the risk of creating other-imposed self-fulfilling prophecies. These occur when a person's expectations govern another's actions (37). In this scenario, the assumptions of others that people with disability have a lower quality of life may lead to them actually having a lower quality of life. It is as important as ever that health-related systems acknowledge and honor the intrinsic worth of all, regardless of factors such as spinal cord injury (24). Issues with ableism also emerge in our second theme, regarding struggling with new challenges.

### Struggling With New Challenges

Participants experienced multiple sources of anxiety and frustration during the pandemic. Several frustrations stemmed from the behaviors of others with people not following guidelines. It has been suggested that, in the general population, the risk of financial loss is a stronger motivator for following or flaunting rules compared to the potential negative health connotations (38). Others not following guidelines represents a key issue for individuals with spinal cord injuries due to their potential inability to take as many precautions themselves, their greater susceptibility of infection, and the potential for their altered physiology to influence which symptoms are displayed delaying the diagnosis of COVID-19 (39). The lack of consideration demonstrated by people not following rules is indicative of person without disability privilege, with people without disabilities only willing to give up ability privileges that they deem as reasonable (25).

A major challenge outlined by participants has been the lack of social interaction. This largely stems from the physical distancing requirement which consequentially has limited the typical movement of individuals. Reduced social interaction could lead to the risk of social isolation and the psychological embodiment of isolation in the form of loneliness (40). The stages of the “Model for Understanding Loneliness” developed by Perlman and Peplau pre-pandemic offers a way of conceptualizing how life during the pandemic could have led to these feelings (41). The precipitating event of the COVID-19 pandemic and requirement to remain physically distanced from others during this time may result in a mismatch between “needed or desired social relations” and the “actual social relations.” This discrepancy, combined with the individuals' cognitive factors, may lead to an experience of loneliness. Both loneliness and social isolation are linked to substantially lower physiological and psychological health (42). This includes higher rates of morbidity, more chronic illnesses, higher depression scores, and less physical exercise (23, 43). The potential health impacts are akin to that of smoking (44, 45). As such, there is a clear need to ensure that adequate social interaction and support is available, along with appropriate resources specifically targeting mental health support. Based on our findings, and other research that has focused on mental health during the pandemic, strategies include ensuring gatekeepers and the public are kept up to date regarding which resources are available and any changes to the usual operations of those services (27). They should also be made aware of potential symptoms to look out for in themselves and others. Telehealth may be a good option for greater outreach and removing the fear of going into health settings, but further research could be needed for its optimal implementation.

### Being Resilient in the Face of a New Normal

Our third theme indicates how participants were able to respond to several changes and challenges thanks in part to their resilience, and augmented by their social support. Resilience can be described as the “process of adapting well in the face of adversity, trauma, tragedy, threats or significant sources of stress” (46). In certain circumstances, enduring previous traumatic experiences can result in the reintegration of self (47). In other words, the event of a spinal cord injury leads to cumulative adversity that develops over time and involves transactions between the individuals' current situation and their past experiences (48). This evolving interplay between personal characteristics, environmental resources, and self-regulatory processes can lead to resiliency-related outcomes. Development in each aspect may foster skills to handle future traumatic events, such as the COVID-19 pandemic. Similar to the current pandemic, a spinal cord injury impacts most aspects of life and living in a subacute rehabilitation facility can be akin to having to quarantine. They have also had to face the barriers imposed by systematic discrimination toward disability, such as having to work harder than people without disabilities for the same roles (49). Although this may have prepared them for the pandemic more than those who have not experienced a spinal cord injury, it reiterates substantial issues of equity.

This study offered the chance to gain a subjective insight into the lives of the spinal cord injury community during the COVID-19 pandemic and how it compared to life pre-COVID-19. We hope that this information can be useful across the spectrum from systemic to individual. Knowing areas of concern or requirement will help legislative bodies direct attention and resources to the necessary areas to facilitate greater quality of life among those with spinal cord injuries. Additionally, gaining an insight into the experiences of the spinal cord injury community will help develop resources that can be broadly disseminated to others that may find the suggestions useful. By drawing upon the mobilities paradigm, we were able to further push the importance of movement in the modern world and the impacts of its restriction. It allowed us to extend our exploration and acknowledge the importance of the movement of goods, services, and information, in addition to the movement of people.

### Limitations

This study has two main limitations, firstly it only captures perspectives early in the pandemic which may limit the breadth of data regarding overall life through the pandemic. Our sample of 22 participants does provide an array of experiences, however, and it provides a clear insight as to how life has changed since pre-COVID-19 and what the initial few months during COVID-19 have been like. A perspective that may have been missed is of individuals with spinal cord injury who have contracted COVID-19 as no one in our sample had. Secondly, as this was part of a larger study, we were unable to sample purposively. Our use of convenience sampling and certain inclusion criteria may reduce how representative our sample is of the population of interest. For example, access to technology is a requirement for inclusion and as only 70% of those with a spinal cord injury may use a computer, the study might miss out on the perspectives of individuals with spinal cord injury who do not have access to technology (50). Of those who did participate, there were some demographic data we did not collect, such as race, which may have been useful.

## Conclusion

In conclusion, this study provided novel insights into the experiences of individuals with spinal cord injury during the COVID-19 pandemic. While the pandemic has presented an array of challenges for individuals with spinal cord injury, it has also presented the opportunity to highlight potential systemic issues that may need addressing. With physical mobility restricted, the movement of goods and information becomes increasingly important. To continue their resilient adjustment to life during the COVID-19 pandemic, our findings emphasize the need for increased dialogue with people in the spinal cord injury community about their ongoing pandemic related needs. Future research could explore comparisons between SCI populations and others or between sub-groups within the SCI population, such as an urban/rural divide. Future research could also continue to explore potential sources and intersectionality of discrimination experienced by individuals with disabilities, such as spinal cord injury, and use the current pandemic as a springboard to promote a change toward greater equity.

## Data Availability Statement

The original contributions presented in the study are included in the article/Supplementary Material, further inquiries can be directed to the corresponding author/s.

## Ethics Statement

The studies involving human participants were reviewed and approved by University of British Columbia and Vancouver Coastal Health (H14-01737). The patients/participants provided their written informed consent to participate in this study. Written informed consent was obtained from the individual(s) for the publication of any potentially identifiable images or data included in this article.

## Author Contributions

ES: conceptualization, methodology, analysis, and writing. WCM, JS, and JB: conceptualization, methodology, and writing. WBM: conceptualization, methodology, analysis, writing, and supervision. All authors contributed to the article and approved the submitted version.

## Funding

This work was supported by WBM New Investigator Award from the Canadian Institutes of Health Research and JB Canada Research Chair in Rehabilitation Engineering Design #230997.

## Conflict of Interest

The authors declare that the research was conducted in the absence of any commercial or financial relationships that could be construed as a potential conflict of interest.

## Publisher's Note

All claims expressed in this article are solely those of the authors and do not necessarily represent those of their affiliated organizations, or those of the publisher, the editors and the reviewers. Any product that may be evaluated in this article, or claim that may be made by its manufacturer, is not guaranteed or endorsed by the publisher.
