# Supplementary material for: The COVID-19 Pandemic Related Lived Experiences of Individuals With a Spinal Cord Injury/Disease
Source: Front Rehabil Sci. 2022 Apr 5;3:834909. doi: 10.3389/fresc.2022.834909 (PMC9397773; doi:10.3389/fresc.2022.834909)
Supplement: Supplementary file 1 [file Data_Sheet_1.pdf]

Interview guide

Hello, my name is _____________ and I would like to start the interview by thanking you for your participation. It is greatly appreciated. The purpose of this interview is to explore and gain a deeper understanding of your experiences and perceptions during the COVID-19 pandemic.

Throughout the interview, you will be asked about your personal experiences. The interview will take approximately 60 minutes. Per the Informed Consent you signed, I just want to remind you that your participation is voluntary. You can stop the interview at any time, if you no longer want to participate. You also do not need to answer any questions which make you feel uncomfortable. During the interview, my main job is to listen to you and your stories. I look forward to hearing about your experiences.

As indicated in the Informed Consent, this session will be video recorded and then transcribed. I will let you know when I will start recording. If your name happens to come up in the interview, it will be removed when the interview is transcribed and replaced with a pseudonym. Do you have any questions for me before we start? I am now going to start recording.

1. Tell me about your spinal cord injury.
   Prompt: How did it occur?
   Prompt: At what level?
   Prompt: What are your capabilities?
2. Tell me what you've learned over the past month during COVID.
3. What has your experience of COVID been like?
   Prompt: Emotionally, physically (exercise), socially.
4. What was a typical day like for you before the COVID quarantine?
   Prompt: What does a typical day look like for you now?
   Prompt: What is the most meaningful part of your day?
   Prompt: Which of these changes do you feel are positive?
   Prompt: Which of the changes do you feel are negative?
5. What are you doing more of?
6. What are you doing less of?
7. What activities are you doing to connect with others?
   Prompt: How would you describe your living situation?
8. What activities are you doing to contribute?
   Prompt: To your personal well-being, to society, to the world?
9. What type of restorative activities are you engaging in?
   Prompt: How do you feel when you are doing your daily activities?
10. How, if at all, has the meaning of the activities you do changes with the advent of COVID?
11. What are some of the biggest challenges you have encountered?
12. What are some of the strategies and supports you have used to overcome them?
13. Knowing the ways you've adjusted to the current situation, what would you recommend to others?
14. What ideas do you have about innovations that could facilitate that change?
    Prompt 1. How, if it all, has your use of social media changed during this time?
    Prompt 2: Describe your use of technology currently.
15. What are your future plans?
    Prompt: Today, this week, monthly, before next interview, for the year

At interview's end:

I am now turning off the recording. I would like to thank you for taking the time to share your experiences. They will be helpful to our research. You will receive your payment as was mentioned previously.
Thank you again. Have a great evening!
